# Supplementary material for: Correlating continuously captured home-based digital biomarkers of daily function with postmortem neurodegenerative neuropathology
Source: PLoS One. 2023 Jun 8;18(6):e0286812. doi: 10.1371/journal.pone.0286812 (PMC10249904; doi:10.1371/journal.pone.0286812)
Supplement: S1 Table — a. Linear regression model showing association between DB composite score and Braak stages (n = 23). b. Linear regression model showing association between DB composite score and Neuritic plaque severity (n = 23). (DOCX) [file pone.0286812.s001.docx]

| **Supplemental Table 1a.** Linear regression model showing association between DB composite score and Braak stages (n=23) | | | | | | |  |  |  |  |
| --- | --- | --- | --- | --- | --- | --- | --- | --- | --- | --- |
| **Variable** | **DF** | **Parameter Estimate** | **Standard Error** | | **t Value** | | | **Pr > \|t\|** | |  |
| Intercept | 1 | 0.19926 | 0.30214 | | 0.66 | | | 0.5171 | |  |
| Braak III/IV vs I/II | 1 | -0.07672 | 0.34005 | | -0.23 | | | 0.8238 | |  |
| Braak V/VI vs I/II | 1 | -0.66569 | 0.42730 | | -1.56 | | | 0.1349 | |  |
| **Supplemental Table 1b.** Linear regression model showing association between DB composite score and Neuritic plaque severity (n=23) | | | | | | |  |  |  |  |
| **Variable** | **DF** | **Parameter Estimate** | | **Standard Error** | | **t Value** | | | **Pr > \|t\|** | |
| Intercept | 1 | 0.39958 | | 0.16507 | | 2.42 | | | 0.0251 | |
| Sparse plaques vs none | 1 | -0.43129 | | 0.25724 | | -1.68 | | | 0.1092 | |
| Moderate/frequent plaques vs none | 1 | -0.90032 | | 0.26955 | | -3.34 | | | 0.0033* | |

* p<0.01; Braak stage I/II group and neuritic plaque none group are the reference groups in table 2a and 2b, respectively.
